# Worms, Fat, and Death: *Caenorhabditis elegans* Lipid Metabolites Regulate Cell Death

**DOI:** 10.3390/metabo11020125

**Published:** 2021-02-23

**Authors:** Marcos A. Perez, Jennifer L. Watts

**Affiliations:** School of Molecular Biosciences, Washington State University, Pullman, WA 99164, USA; marcos.perez@wsu.edu

**Keywords:** apoptosis, ferroptosis, polyunsaturated fatty acids

## Abstract

*Caenorhabditis elegans* is well-known as the model organism used to elucidate the genetic pathways underlying the first described form of regulated cell death, apoptosis. Since then, *C. elegans* investigations have contributed to the further understanding of lipids in apoptosis, especially the roles of phosphatidylserines and phosphatidylinositols. More recently, studies in *C. elegans* have shown that dietary polyunsaturated fatty acids can induce the non-apoptotic, iron-dependent form of cell death, ferroptosis. In this review, we examine the roles of various lipids in specific aspects of regulated cell death, emphasizing recent work in *C. elegans*.

## 1. Introduction

Regulated cell death is vital for proper development, tissue homeostasis, and as a response to DNA damage [1,2]. Molecular processes required for regulated cell death are often disrupted during diseases states, such as cancer and neurological disorders [3,4]. Studies of the mechanisms of apoptosis, the first described form of regulated cell death, were spearheaded by genetic studies in the Horvitz lab using the roundworm *Caenorhabditis elegans* (*C. elegans*) [5,6]. This early work elucidated the core apoptotic pathway consisting of *ced* genes encoding APAF1, BCL2, and BH3-only proteins. Since then, thousands of papers have been published elucidating mechanistic details of apoptosis, including the roles of conserved proteins involved in the engulfment and degradation of cells [7,8].

Cells die by accident or by genetically regulated pathways. Classic necrosis, accidental cell death due to severe stress, occurs when a cell dies due to swelling and lysis of the plasma membrane [9]. In contrast, regulated cell death is a process in which cells carry out a series of orchestrated biochemical steps leading to cellular demise. The most common and best studied regulated cell death pathway is apoptosis, which can be initiated during development by pro-death signals or by a lack of pro-survival signals, and is also initiated extrinsically in response to DNA damage and environmental stressors. Recently, several non-apoptotic pathways of regulated cell death have been reported [1]. These include the immunological and inflammatory forms of cell death such as pyroptosis [10,11], necroptosis [12,13,14,15], and NETosis [16,17]; the loss of cell-matrix interaction from detachment of the extracellular matrix form of death, anoikis [18,19]; iron-dependent cell death, ferroptosis [20,21]; the cell-eating and cannibalistic forms of cell death phagoptosis [22,23], entosis [8,24] and linker-cell type death [1,25,26]; the glucose-dependent overproduction of ROS form of cell death, parthanatos [27,28,29]; and upon disequilibrium of homeostasis, the self-eating form of cell death, autophagy [8,30,31,32]. Each regulated cell death pathway displays unique biochemical characteristics. As more studies emerge, many biochemical connections between the various death pathways have become apparent and are still being elucidated [33]. 

In *C. elegans*, apoptosis occurs during embryonic development as well as in the adult germ line [34]. In the germ line, DNA damage induces apoptosis, while damaged somatic cells die by necrosis. *C. elegans* has contributed to mechanistic insight into many non-apoptotic death processes. Autophagy is well described and shown in *C. elegans* to occur through dietary restriction [31,35] in germline-less animals [36,37] and these modulate aging through this process. Necrosis was found to occur in neurodegenerative contexts through ionic imbalances and heat-induced stress [38,39,40] as well as in instestinal bacterial pathogenesis [40,41]. Entosis was recently found to be involved in linker-cell death in male development, where a migratory linker cell undergoes death to establish gonad-cloaca fusion prior to mating [42]. Finally, ferroptosis is induced by dietary fatty acids in the germ line [43] and occurs in somatic cells during aging [44]. These new findings are thoroughly reviewed below. Although *C. elegans* studies have been vital to many discoveries in the field of cell death, one limitation is the lack of specialized immune cells to study cell death pathways specific to immunity and inflammation, such asnecroptosis, pyroptosis, NETosis and parthanatos. 

Lipids are important structural components in the cell and are powerful signaling molecules for many processes, including inflammation, stress responses and development. Lipid metabolites are essential for cellular function in living cells and they can drive cell death processes as well. A recent review summarizes the mechanistic contributions of lipids to apoptosis, necroptosis, and ferroptosis in mammals [45]. Our review focuses on aspects of apoptosis and ferroptosis in which *C. elegans* studies have provided insights into the roles of lipid metabolites in regulated cell death pathways.

### C. elegans Lipid Composition

**Figure 1:**
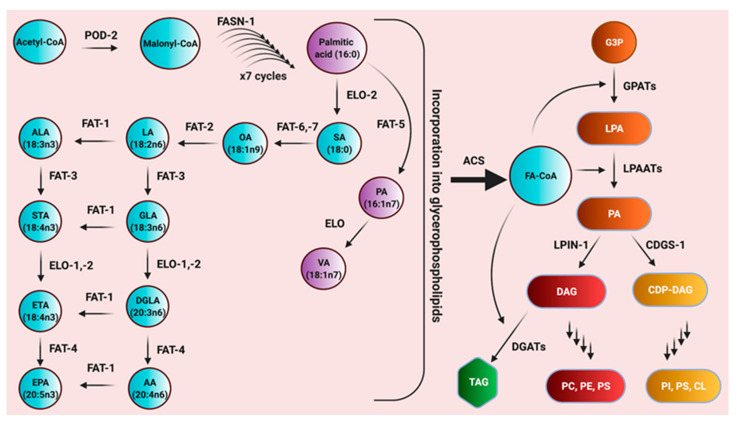
The pathway for polyunsaturated fatty acid biosynthesis and the incorporation of fatty acids into glycerophospholipids in *C. elegans*. Enzyme names are abbreviated in black over the arrows. POD-2, acetyl-CoA carboxylase; FASN-1, fatty acid synthase; ELO, elongase, FAT-5 ∆9 desaturase; FAT-6/-7, ∆9 desaturases; FAT-2, ∆12 desaturase; FAT-1, omega-3 desaturase; FAT-3, ∆6 desaturase; FAT-4, ∆5 desaturase; ACS; acyl CoA synthetases; GPATs; glycerol-3 phosphate acyl transferases; LPAATs, lysophosphatidic acid acyl transferases; LPIN-1, lipin; CDGS-1, CDP-diacylglycerol synthase; DGATs; diacylglycerol acyl transferases. The metabolites in the pathways are enclosed in bubbles. SA, stearic acid; PA, palmitoleic acid; VA, vaccenic acid; OA, oleic acid; LA, linoleic acid; ALA, alpha linolenic acid; GLA, gamma linolenic acid; DGLA, dihomo gamma linolenic acid; AA, arachidonic acid; STA, stearidonic acid; ETA, eicosatetraenoic acid; EPA, eicosapentaenoic acid; FA-CoA, fatty acids conjugated to CoA; G3P, glucose-3 phosphate; LPA; lysophosphatidic acid; PA, phosphatidic acid; DAG, diacylglycerol; CDP-DAG, cytidine diphosphate diacylglycerol; TAG, triacylglycerols; PC, phosphatidylcholine; PE, phosphatidylethanolamine; PS, phosphatidylserine; PI, phosphatidylinositol; CL, cardiolipin. Adapted from [52].

The roundworm *C. elegans* is a multicellular organism widely used to explore a range of biological questions in the context of a sexually reproducing organism. Much effort in recent years have been placed on understanding how lipids influence metabolic processes in development, aging, and death [46,47,48,49]. *C. elegans* phospholipids contain a range of fatty acids, including saturated fatty acids, monounsaturated fatty acids (MUFAs), and polyunsaturated fatty acids (PUFAs). While core lipid metabolism pathways are conserved in *C. elegans* and other animals, the worm is unusual compared to most animals because it can synthesize long-chain omega-6 and omega-3 PUFAs *de novo* from acetyl-CoA or from dietary lipid precursors from their *E. coli* diet [50,51,52]. PUFA formation relies on a family of fatty acid desaturases and elongases, each with specific substrate specificities to produce a range of 18- and 20-carbon long chain PUFAs with 2–5 double bonds (Figure 1) [50,51]. The presence of plant-like ∆12 and omega-3 desaturase activity obviates the need for dietary essential fatty acids required by mammals and most other animal species. Using *C. elegans*, PUFAs have been studied in many contexts from reproduction and development [53,54,55,56,57,58] to stress, aging and cell death [43,59,60,61,62,63] and these studies have been extensively reviewed [64,65].

Lipid composition in *C. elegans* has been quantified by various lipidomic tools, including gas chromatography-mass spectrometry to identify fatty acid composition [51], thin-layer chromatography to identify the major lipid classes [66,67], and liquid chromatography mass spectrometry to identify specific lipid species [68]. Furthermore, stable isotope studies have been used to distinguish between dietary and *de novo* synthesized fatty acids and to study the kinetics of lipid turnover in the whole organism. These studies demonstrated that greater than 80% of *C. elegans* fatty acids in phospholipids and neutral lipids derive from dietary or modified dietary fats obtained from the *E. coli* diet [69]. Remarkably, kinetic assays demonstrated that 2.4% of storage lipids and 4.7% of membrane lipids are turned over every hour, resulting in nearly complete turnover of animal lipids every day [70].

In *C. elegans*, dietary and *de novo* synthesized fatty acids become incorporated into neutral lipids, membrane sphingolipids, and membrane glycerophospholipids (Figure 1). The most abundant membrane phospholipids are phosphatidylcholines (PC), phosphatidylethanolamines (PE), phosphatidylserines (PS), phosphatidylinositols (PI), and cardiolipins [52,68,70]. These lipids are important structural components of cells and are arranged asymmetrically in membranes, with varying compositions in the inner and outer leaflets of the plasma membrane and organelles [71]. Aside from their structural roles, phospholipids and their derivatives are powerful signaling molecules that play important roles in many biological processes, including cell death. 

## 2. Apoptosis

Apoptosis was first characterized genetically in *C. elegans* and is an important process for development in physiological apoptosis and in stress-induced apoptosis [72,73]. In the worm, there are two distinct modes of apoptosis: developmental cell death, that occurs in the embryo [74] and post-embryonically [75]; and germ cell death, which occurs in the gonad as part of normal gonadal development, and also occurs as a result of exogenous exposure to DNA damaging agents, such as ionizing and ultraviolet radiation, as well as alkylating agents [76,77]. Both of these cell modes of apoptosis can be visualized in the animals via differential inference contrast (DIC) and appear “button-like” from the refraction [73]. Morphological features of apoptosis includes chromatin condensation and fragmentation, mitochondrial damage via the loss of the membrane potential, and asymmetry of the membrane phospholipids that signal cell death [34,78]. Although apoptosis plays an important role in developmental processes in the worm, as well as in other species, it is also activated through DNA damage, oxidative stress and other exogenous stressors if the cell cannot overcome this onslaught [79,80]. 

In the worm, apoptosis is activated by the core apoptosis machinery. The core pathway includes EGL-1, a pro-apoptotic BH3-only-domain protein, CED-9/BCL2, CED-4/APAF1-like protein, and CED-3/CASP3. Apoptosis occurs in several stages. The specification phase determines which cells will live or die. During the activation phase, the caspase is activated and acts to degrade a large number of cellular proteins leading to the execution phase, where cells undergo shrinkage, chromosomal condensation, DNA fragmentation and blebbing. Finally, dead cells are removed by neighboring cells by phagocytosis [81]. The mechanisms of apoptosis during embryonic developmental and in adult germ cells have been extensively reviewed in other sources [34,82], and the roles of lipids in apoptosis are highlighted here.

### 2.1. Phosphatidylserine in Signaling Execution in Apoptotic Cells

**Figure 2:**
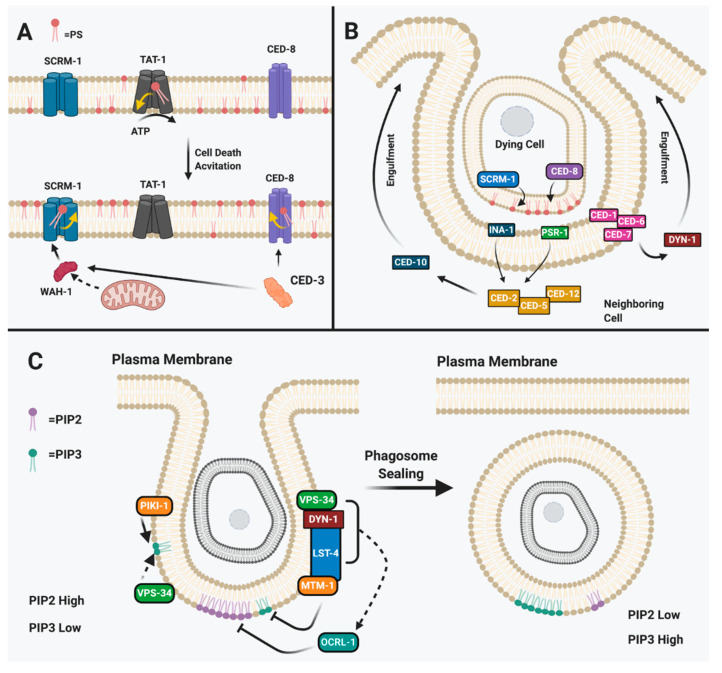
Phospholipids in *C. elegans* apoptosis. (**A**) Phosphatidylserine (PS) plays a role in the specification of the apoptotic cell. TAT-1 is involved in regulating asymmetry of PS in the plasma membrane and this activity is reduced upon cell death activation. CED-3 proteolytically activates WAH-1, which acts to translocate to SCRM-1 and increase the number of PS molecules in the outer leaflet. CED-3 also proteolytically activates CED-8 in the plasma membrane to translocate PS from the inner leaflet to the outer leaflet. Adapted from [93]. (**B**) During the execution phase, the dying cell is engulfed by the neighboring cell in *C. elegans*. The PS serves as a “eat me” signal to the neighboring cell which will engulf the apoptotic cell through two arms of the engulfment pathway: the CEDl-1/CED-6/CED-7/DYN-1 pathway and the CED-2/CED-5/CED-12/CED-10 pathway leading to elongation of pseudopodal arms around the corpse. Adapted from [34]. (**C**) PIP3 accumulation is required to consume the dead cell in phagosomal sealing. Phosphotidylinositol-4,5-bisphosphate (PIP2) accumulates in unsealed phagosomes while phosphotidylinolsitol-3-phosphate (PIP3) accumulates in sealed phagsosomes. Adapted from [94].

Phospholipids are asymmetrically distributed throughout the plasma membrane in eukaryotes, with phosphatidylethanolamines (PE) and phosphatidylserines (PS) generally localized to the inner leaflet and phosphatidylcholines (PC) and sphingolipids mainly localized to the outer leaflet [83,84]. PS itself is involved in the execution phase of apoptosis, where it is translocated from the inner leaflet to the outer membrane of the plasma membrane and acts as a conserved “eat me” signal (Figure 2A) [85,86,87,88,89]. In *C. elegans*, the phagocytosis of these dead cells is accomplished by neighboring cells because they do not possess dedicated macrophages to perform this task [34,74,90,91]. Various lipid transporters are involved in the externalization of PS in the execution phase, which can be measured by GFP-tagged Annexin V, MFG-E8 and lactadherin [34,92]. 

Scramblases are bidirectional, non-specific, ATP-independent, calcium-dependent phospholipid transporters [95]. Annexin V staining in the dissected gonads of worms revealed that PS is externalized by plasma membrane-bound scramblase, SCRM-1/PLSCRs [92,93,96]. However, mutants of *scrm-1* exhibit only a partial reduction in PS externalization in germ cells undergoing apoptosis, suggesting another factor regulating this activity. This factor was later identified as the mitochondrial-bound apoptosis inducing factor 1, WAH-1/AIF1, which is released from the mitochondria through proteolytic cleavage by CED-3 [92,93]. This mitochondria-to-plasma membrane PS externalization signaling acts in a similar manner within mammalian systems in cell corpse clearance by human macrophages, showing a conserved feature of PS externalization through this mechanism [97]. In embryos, CED-8/XKR8 is activated by CED-3 to externalize PS [98,99]. Activation of CED-8 by CED-3 generates acCED-8, a proapoptotic protein with a carboxy-terminal cleavage product that can externalize PS in apoptotic cells and ectopically expose PS in live cells, a feature conserved in mammalian systems [98,100]. 

Externalization of PS is controlled by membrane-bound TAT-1/ATPases and in either *tat-1* knockdown or mutants, PS is ectopically expressed on the outer leaflet of the plasma membrane in living cells, suggesting a regulation of this activity in apoptotic cells [101,102]. This was confirmed in mammalian systems where ATP11C and CDC50A possess phospholipid translocase activity [103]. Together these proteins act to maintain PS in the inner membrane leaflet unless they are inactivated by caspase, resulting in PS exposure on the outer membrane leaflet [103]. Interestingly, *tat-1* mutants can also accumulate large endolysosomal vacuoles with PS externalization in the intestine suggesting that maintenance of PS asymmetry plays a role not only in apoptosis, but also endosomal trafficking [104,105]. Though the mechanism of this process is still poorly understood, a recent analysis of *C. elegans* mutants from the million mutants project identified specific motifs of TAT-1, PISL and DKTGT that play a functional role in the flippase activity, and that these domains are conserved in mammalian systems [106]. Finally, TAT-1 activity is controlled by PSR-1/JMJD6 and CED-1/LRP1 as loss of function mutants of either of these receptors lead to unengulfed PS externalized living cells [107,108]. PS is therefore a signal for these two receptors that are involved in different pathways of engulfment. 

Ectopic exposure of PS on living cells serves the purpose of triggering engulfment of dying cells, and in *C. elegans* neighboring cells can do this through two redundant pathways that have been well-characterized (Figure 2B). The first pathway involves the receptor CED-1 and an ABC transporter, CED-7/ABCA1, that activate downstream CED-6/GULP1 and DYN-1/DYN2 for phagocytosis of apoptotic cells [34,91,109,110,111]. The other pathway involves PSR-1, which upon interacting with PS on the apoptotic cell activates a complex containing CED-2/CRKII, CED-5/DOCK180, and CED-12/ELMO. This complex then activates the GTPase, CED-10/RAC1, leading to pseudopodal elongation from actin skeleton rearrangement to engulf the apoptotic cell [34,76,108,109,111,112,113]. Interestingly, Neumann et al. [114] found that PSR-1 can function in contexts where PS externalization has been shown to act as a “save me” signal in the regeneration of injured axons through the CED-1/CED-6/CED-7 pathway, suggesting alternative roles of PS outside of cell death. Both pathways eventually lead to the sealing of the phagocyte and the cell corpse is consumed [34,94,115]. This process is mediated by various factors involved in phosphatidylinositol (PI) signaling. 

### 2.2. Phosphotidylinositol in Consuming the Apoptotic Corpse

Phosphoinositides, phosphorylated derivatives of PI, are powerful signal transduction molecules involved in many cell biological processes [116,117]. In apoptosis, phosphoinositides act as signaling molecules and also as localization cues, with various species dynamically localizing to specific membrane regions during cell engulfment [118]. Specifically, phosphatidylinositol-4,5-bisphosphate (PIP2) accumulate in unsealed phagosomes and phosphatidylinositol-4,5,6-bisphoshphate (PIP3) accumulate in sealed phagosomes and are important in the final encapsulation of the cell corpse (Figure 2C). Studies in *C. elegans* demonstrated that the PIP3 accumulates in two waves, first during formation of the phagosome and a second wave lasting until digestion of the cell is completed [119,120]. Using transgenic lines expressing genetically encoded PI sensors, Lu et al. found that the dynamic localization of the phosphoinositide species is regulated by two kinases, PIKI-1/PIK3C2A and VPS-34/PIK3C3 and one phosphatase, MTM-1/MTM1 [119,120]. Both kinases are recruited to the maturing phagosome by the DYN-1 GTPase, where DYN-1 binds to VPS-34 [119,121]. 

The kinases have slightly different ways of modulating PIP3 levels: PIKI-1 works in conjunction with MTM-1 to regulate PIP3 in sealing of the phagosome, while VPS-34 itself produces PIP3 during sealing of the phagosome [120]. DYN-1 and the SNX9 family protein LST-4/SNX9 are key regulators of phagosomal sealing and are recruited to the site of unsealed phagosomes and recruitment is influenced by levels of PIP2, PIP3 and MTM-1 [94,119,120]. Interestingly, Abdu et al. [122] showed that LST-4 is also involved in endocyte vesicle scission in the maintenance in progenitor germ cells (PGC). Specifically, they found LST-4 and CED-10 act together in the cannibalism of PGC lobes from the association with endodermal cells by scission of the lobe into the endodermal cell through recruitment of actin and dynamin as they migrate through the rachis. Loss of function mutants of either *lst-4* or *ced-10* led to the persistence of lobe necks in the cannibal endodermal cell [122]. They also showed that mitochondria were abundant in the lobes, and suggested to act as a pruning mechanism of high reactive oxygen species (ROS) generated by the mitochondria as the PGC migrate through the rachis [122]. 

Recently, Lee et al. [42] showed a similar type of mechanism of the lobe scission of linker cells to facilitate the fusion of gonad-to-cloaca for maturation in male worms and claim that this occurs through entosis. Using a reporter in the cell that expresses phospholipase C delta with GFP conjugated to the PH domain (GFP-PLC**δ**-PH) to increase PIP2, they showed that lobe scission for entotic death was inhibited [42]. Given levels of PIP2 reduces lobe scission, perhaps LST-4 may play a role in entotic cell death, as well as in phagosome formation in apoptosis. For the phagosome to seal, PIP2 levels are depleted and this is accomplished by OCRL-1/OCRL and is a conserved mechanism in mammals and worms [120,123,124]. The mechanism of how OCRL-1 is recruited is not well understood and merits further investigation.

## 3. Ferroptosis

Ferroptosis is a caspase-independent form of regulated cell death involving oxidized lipids and redox active iron [20,21]. Ferropotsis is a mechanism for the killing of malignant cells, but it also contributes to cell death during disease states, especially in tissues with high oxidative capacity, such as neurons, heart, kidneys, and lungs [20,125,126,127,128]. Early studies of ferroptotic mechanisms focused on the lethal mechanism of small molecules, such as erastin, which depletes glutathione (GSH), and RSL3, which covalently inactivates glutathione peroxidase 4 (GPX4). These compounds can trigger cell death in cancer cell cultures [20,21,129,130]. Importantly, radical trapping antioxidants, such as ferrostatin-1, vitamin E, and liproxstatin-1 protected cells from death, as did the chelation of iron [20,21,126,131]. Thus, early studies confirmed that iron-dependent oxidized cellular components contribute to the cell death.

GPX4 converts potentially toxic lipid peroxides to more benign lipid alcohols [21,126,130,132,133], implicating oxidized lipids in ferroptosis. PUFAs with multiple double bonds are highly susceptible to oxidation. PUFAs are especially susceptible to peroxidation over their double bonds, where the hydroxy free radical can abstract a proton from the bis-allylic position generating a carbon radical and this can react with other PL-PUFAs leading to a chain reaction of lipid peroxide propagation [134,135]. A major ferroptosis regulator in mammals is the acyl-CoA synthetase long chain family member 4 (ACSL4), the acyl-transferase enzyme responsible for the insertion of PUFAs into membranes [21,136]. Roles for oxidized PUFAs in promoting ferroptosis were revealed in lipidomic studies that identified oxidized PE lipids in cancer cells treated with RSL3 [136,137]. Specifically, peroxides of omega-6 PUFAs, arachidonic acid (AA, 20:4n-6) and adrenic acid (AdA, 22:4n-6) were enriched and lipoxygenase enzymes were predicted to be required for the peroxide formation [129,137,138].

### 3.1. Dietary Polyunsaturated Fatty Acid Induction of Ferroptosis in C. elegans Germ Cells and Cancer Cells

Dietary studies in *C. elegans* revealed that omega-6 PUFAs, dihomo-gamma-linolenic acid (DGLA; 20:3n-6) and, to a lesser extent, arachidonic acid (AA, 20:4n-6) caused germ cell death and sterility in *C. elegans*. Only DGLA and high doses of arachidonic acid induced the germ cell death, there was no death noted in studies in which linoleic acid (LA, 18:2n-6) or omega-3 fatty acids, such as alpha-linolenic acid (ALA, 18:3n-3) or eicosapentaenoic acid (EPA, 20:5n-3) were supplemented [55]. While gonads showed increased numbers of apoptotic corpses, diet-induced germ cell death still occurred in the apoptosis-defective *ced-4* mutants, demonstrating that this process was caspase-independent [55,59]. Genetic analysis demonstrated that mutant strains deficient in n-6 PUFA biosynthesis, especially FAT-2 (∆ 12 desaturase) and FAT-3 (∆ 6 desaturase) are resistant to DGLA-induced sterility [43,55]. 

To demonstrate that DGLA-induced sterility is caused by ferroptosis, Perez et al. used ferrostatin-1, along with vitamin E, to demonstrate that radical trapping antioxidants protected the worms from dietary DGLA [43]. As has been shown in mammalian ferroptosis, iron chelation protects germ cells from DGLA-induced germ cell demise, while a strain carrying a glutathione peroxidase mutation led to increased sensitivity to DGLA. Like in mammalian systems, worm glutathione phospholipid hydroperoxidases play a key role in the inhibition of phospholipid peroxidation and ferroptosis [43,130,132,139].

**Figure 3:**
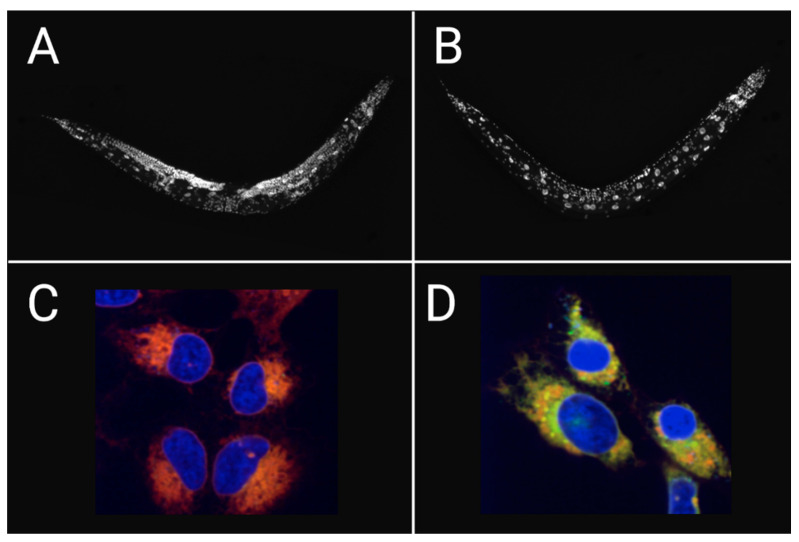
Dietary DGLA induces ferroptosis in *C. elegans* and human cancer cells. Worms are stained with DAPI showing (**A**) healthy germ cells in untreated worms and contrasts (**B**) sterile worms depleted of germ cells after DGLA-induced ferroptosis. HT-1080 cancer cells co-treated with Hoechst and the lipid peroxidation probe, C11-BODIPY, and further treated (**C**) without DGLA (red; non-oxidized) and (**D**) with DGLA (yellow; oxidized) that undergo ferroptosis. Adapted from [43].

Importantly, dietary DGLA-induced ferroptosis is not just observed in nematodes, supplementation of DGLA was sufficient to kill human cancer cells. Visualization of lipids using the molecular probe C11-BODIPY revealed that DGLA-treated cells showed increased levels of lipid peroxides, which was rescued by Fer-1 (Figure 3) [43,140]. Lipidomic analysis of DGLA-treated worms and cancer cells demonstrated that DGLA accumulates in PC and PE phospholipids. Thus, DGLA alone is sufficient to induce ferroptotic cell death, even in the presence of intact antioxidant responses, including glutathione peroxidase activity [43]. 

### 3.2. Dietary Monounsaturated Fatty Acids Protect Cells from Ferroptosis

While omega-6 PUFAs can trigger ferroptosis, studies in both mammalian cells and *C. elegans* show that MUFAs, especially oleic acid (OA; 18:1n-9), can inhibit ferroptosis. Magtanong et al. [141] found that OA is incorporated into the plasma membrane of mammalian cells via ACSL3 (acyl-CoA synthetase long chain family member 3). Here, MUFAs can displace more oxidizable AA species, leading to ferroptosis-resistance. Similarly, *C. elegans* raised on a diet of OA in addition to DGLA were fertile and showed very little germ cell death [43]. Lipidomic analysis showed that OA displaced the omega-6 PUFAs DGLA and AA, but not the omega-3 PUFA EPA [43]. Furthermore, genetic manipulations, such as the *C. elegans fat-2* mutants, which have a higher OA content and lower C20 PUFA content, were more resistant to dietary DGLA-induced ferroptosis [43]. 

MUFAs have generated great interest because of the association of diets rich in MUFAs with positive health outcomes. For example, incorporating more MUFAs into the diet has been suggested to play a role in reducing the onset and severity of cardiovascular disease, such as atherosclerosis, by displacing saturated fatty acids that lead to the development of atherosclerotic plaques [142,143]. In *C. elegans*, MUFA synthesis correlates with increased lifespan and healthspan [46]. In the context of ferroptosis, oxidizable PUFAs thought to contribute to cell death in neurodegenerative and cardiovascular diseases could potentially be mitigated by ferroptosis inhibitors administered in conjunction with dietary MUFAs [141,144,145]. 

### 3.3. Iron, Ferroptosis and Aging

Iron is important in many biological processes and is a significant contributor to ferroptosis. Iron plays a key role in lipid peroxidation propagation because ferrous iron catalyzes the Fenton reaction, in which hydroxyl free radicals are generated [146]. These free radicals can then damage macromolecules in the cell, including polyunsaturated fatty acids [134]. An alternate, or additional role for iron in promoting ferroptosis is its role as a cofactor for lipid oxidizing enzymes, such as lipoxygenases and CYP450s [60,138,147,148]. Thus, iron could be essential for the enzymatic reaction responsible for the initiation of ferroptosis by dietary omega-6 fats, as well as contributing to the propagation of lipid peroxides throughout membranes. Ferroptosis can be inhibited by iron chelation in mammalian cell lines and in *C. elegans* [20,21,43]. Additionally, increasing intracellular labile iron in mammalian cells through different mechanisms increases susceptibility to ferroptosis [20,149,150,151]. In *C. elegans,* increasing cellular iron in worm strains carrying mutations in the ferritin gene led to increased susceptibility to DGLA-induced ferroptosis [43]. The physiological role of iron in ferroptosis has been suggested to be involved in tumor survival, where iron transport is reduced from transferrin receptors, leading to a reduction in iron uptake and reduced ferroptosis [21,152,153].

Iron-mediated lipid peroxidation from has been associated with aging, especially in the context of neurodegenerative diseases, such as Alzheimer’s and Parkinson’s disease, where iron is no longer able to be stored efficiently [154,155]. In *C. elegans*, iron has been found to be dysregulated in senescent worms, where ferritin was less capable of sequestering ferrous iron [156]. Recently, Jenkins et al. [44] found iron dysregulation in aging *C. elegans* led to ferroptosis of somatic cells, particularly intestinal cells. This cell death occurred approximately four days before organismal death, contributing to tissue degeneration and frailty. As the worms aged, increased ferrous iron was associated with reduced glutathione levels. Treatment with an iron chelator or with the lipophilic radical trapping antioxidant, liproxstatin-1 reduced frailty during aging and greatly increased lifespan [44]. Furthermore, although the exact lipid species were not investigated, malondialdehyde (MDA) and 4-hydroxynonenal (4-HNE), common lipid peroxidation end products, accumulated in aging worms and iron chelation and Lip-1 treatment ameliorated those peroxide end products [44]. It would be interesting to investigate whether specific dietary lipid species, such as DGLA, contribute to frailty and aging in *C. elegans*.

### 3.4. Ether Lipids in Ferroptosis

Ether lipids are a class of glycerolipids that occur in two groups alkyl-ether and alkenyl-ether lipids (the latter also known as plasmalogens) and are different from glycerphopholipids in that the sn1 position contain an ether instead of an ester bond [157,158]. This ether bond is more rigid than an ester bond, thus ether lipids play a structural role in membranes and in signaling complexes. Ether lipid synthesis begins in peroxisomes, starting with the glycolysis intermediate dihydroxyacetone phosphate (DHAP). Once in the peroxisome, DHAP undergoes acylation at the sn1 position by glyceronephosphate O-acyltransferase (GNPAT) and then alkylation by alkylglycerone phosphate synthase (AGPS) [159]. Further processing of the alkyl-DHAP occurs in the endoplasmic reticulum, where it matures into a functional ether lipid, or is further desaturated at the sn1 position into a plasmalogen [160]. Ether lipids are abundant in several tissues, including the brain, the kidney, and the heart [161]. Ether lipids, especially plasmalogens, have also been shown to be decreased in brain tissues in patients with Alzheimer’s and Parkinson’s disease [162,163]. Ether lipids deficiency also occurs in peroxisomal congenital disorders such as Rhizomelic chondrodysplasia punctata (RCDP) [161,164].

**Figure 4:**
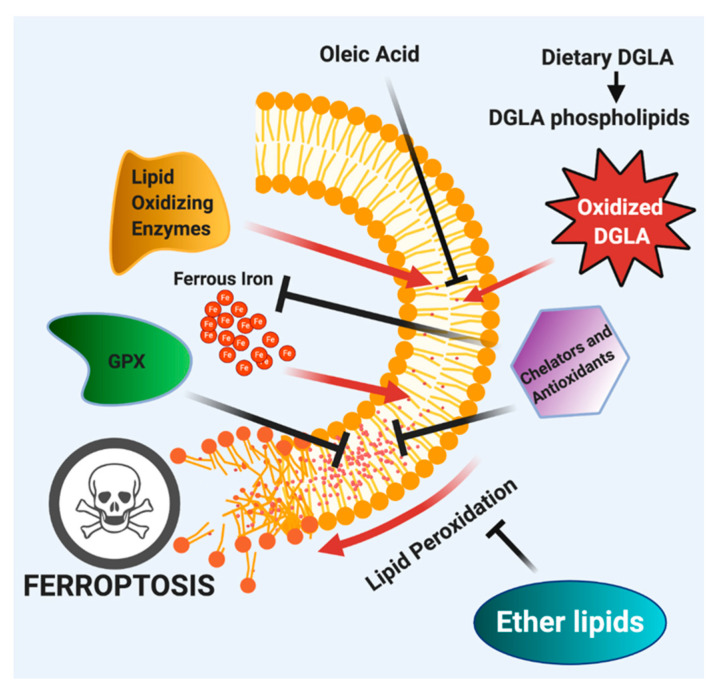
Exogenous DGLA induces ferroptosis by accumulation of lipid peroxides and epoxides of DGLA and further exacerbated by ferrous iron and lipid oxidizing enzymes. This activates lipid repair pathways, including GPXs and can be inhibited by iron chelators and radical trapping antioxidants. Finally, ether lipids play a protective function in DGLA-induced ferroptotic cell death by inhibiting lipid peroxidation. Adapted from [43].

Perez et al. found that *C. elegans* mutants lacking the gene encoding the rate-limiting step of ether lipid biosynthesis, AGPS, encoded by *ads-1* in the worm [67] were more susceptible to DGLA-induced ferroptosis than wild type [43] and also sensitive to oxidative stress in general [67]. Furthermore, protection from DGLA-induced ferroptosis was conserved in mammalian systems, as cancer cells co-treated with ZINC-69435460 (AGPS inhibitor) and DGLA were significantly sensitized to cell death, even more so than DGLA treated cancer cells alone [43]. Thus, ether lipids play a protective role in ferroptosis induced by dietary fats. This finding agrees with proposed roles for ether lipids as oxidative “sinks”, specifically plasmalogens, in which oxidation of the enol ether double bond leads to the formation of a non-reactive aldehyde, essentially put the brakes on the lipid peroxidation cascade (Figure 4) [165,166].

By contrast, Zou et al. found that depletion of AGPS in cancer cells using CRISPR/Cas9 technology led to resistance to ferroptosis [167,168]. In this study, cancer cells depleted of the persoxisomal genes required for the first steps of ether-lipid biosynthesis, as well as an ER enzyme required for a later stop of ether-lipid biosynthesis, showed reduced ferroptotic cell death. This reduction of ferroptosis occurred in vitro as well as in vivo when the cells were implanted into immunocompromised mice. Delivering various species of ether lipids to cells using lipophilic nanoparticles revealed that ether lipids species containing PUFAs, but not those containing MUFAs, were responsible for the pro-ferroptotic effects of ether lipids [167]. 

These contrasting findings might be explained by differences in ether lipid composition of *C. elegans* and mammals. Unlike mammals, *C. elegans* ether lipids are only associated with PE and are not detectable in PC species. Approximately 20% of PE lipids are ether-linked lipids, with PUFAs esterified to the sn2 position in approximately 40% of ether lipids, with the rest containing a MUFA or a saturated fatty acid [67]. In ether-lipid deficient mutants, the whole-animal content of PUFAs is not changed compared to wild type, although the worms exhibit higher levels of saturated fatty acids and lower levels of MUFAs [67]. In the human brain, 30% of PE lipids are plasmalogen ether lipids [169] and in cardiomyoctes, 50% of phospholipids in the sarcolemma are plasmalogen ether lipids [170]. Thus, ether lipids are more abundant and contain a high portion of cellular PUFA in mammalian cells than in *C. elegans*. Therefore, inhibiting ether lipid biosynthesis is predicted to considerably inhibit PUFA content of membranes in mammals, while ether lipid depletion decreases MUFA content in *C. elegans*. With ether lipids being such a large reservoir of PUFAs in mammalian cells, the finding that ether lipid synthesis is pro-ferroptotic might be analogous to the pro-ferroptotic role of endogenous synthesis of PUFAs in *C. elegans*.

## 4. Conclusions and Future Directions

*C. elegans* is an indispensible workhorse in the fields of genetics, molecular and cell biology, and recently metabolism. Studies in the nematode have contributed greatly to the field of cell death. Features of apoptosis in mammals and *C. elegans* are summarized in Table 1. Future challenges in the cell death field include identifying physiological roles for ferroptosis. The induction of ferroptosis in germ cells, but not somatic cells, hints at a physiological function of ferroptosis in protecting the most precious cells in an organism from reactive oxygen species and preventing cells with damaged membranes or DNA from becoming gametes. Further studies in *C. elegans* will unravel the tissue-specific mechansims of ferroptosis induction, as well as roles for dietary fats in inducing ferroptosis in mammalian species.

## Figures and Tables

**Table 1:** Summary of Apoptosis and Ferroptosis Mechanisms in Mammals and *C. elegans.*

**Apoptosis**		
**Feature**	**Mammals**	***C. elegans***
**Phosphotidylserine** **presentation as** **“eat me signal”**	Inactivation of ATP11C and CDC50A activity promotes PS exposure and cell death [103].	Inactivation of TAT-1 leads to PS exposure and cell death [98,101,102].
	Activation of PLSCR1 via translocation of AIF from mitochondria [97].	Translocation of WAH-1 from CED-3 cleavage for SCRM-1 activation [92,96].
	Activation of XRC8 via Caspase 3 leads to PS exposure [100].	Activation of CED-8 via CED-3 leads to PS exposure [98,99].
**Corpse engulfment** **via phosphotidylserine**	LRP1 associates with ABCA1 to activate GULP and further activates Dyn2 for actin reorganization and elongation of cell around apoptotic corpse [111].	CED-1 associates with CED-7 further activating CED-6 for DYN-1 associated pseudopodal elongation around apoptotic corpse [91,110].
	CRKII complexes with DOCK180 and ELMO to activate RAC for cytoskeletal rearrangement and elongation of pseudopodal arms around apoptotic cell [111].	CED-2 complexes with CED-5 and CED-12 to activate downstream CED-10 for elongation of pseudopodal arms for engulfment of apoptotic corpse [76,108,112,113].
**Phosphotidylinositol in** **corpse consumption**	Phagosome sealing involves many factors, including the complex of PIK3C2A, PIK3C3, and MTM1 that recruit SNX9 and DYN1; this further recruits OCRL to seal phagosome by increasing PIP2, the phospholipid type that accumulates in sealed phagosomes [116,117,123].	Phagosome sealing involves complexing of PIKI-1, VPS-34, and MTM-1 that recruits LST-4 and DYN-1; this further recruits OCRL-1 to increase PIP2 that accumulates for sealing [119,120].
**Ferroptosis**		
**Feature**	**Mammals**	***C. elegans***
**Requirement of oxidized** **ω-6 polyunsaturated fatty acids**	Oxidized PE-AA, PE-AdA and DGLA act as executioners of ferroptosis in cancer cells [43,136,137,138].	Oxidized DGLA, and to some extent AA, induce ferroptosis of germ cells [43,55,59,60].
**Monounsaturated fatty acids** **in protection from ferroptosis**	Exogenous OA is incorporated into the plasma membrane via ACSL3 to promote a ferroptosis-resistant membrane [141].	Worms fed OA are rescued from dietary DGLA-induced ferroptosis of germ cells [43].
**Requirement of** **iron in ferroptosis**	Cancer cells are more resistant to ferroptosis via downregulation or transferrin receptors reducing iron intake involved in the and are rescued from ferroptosis when treated with iron chelation reducing substrate for the fenton reaction [20,151,152]. Iron also acts as a cofactor for lipid oxidizing enzymes to generate ferroptosis initiators [138,147,148].	Worms fed DGLA in conjunction with iron chelators are rescued from germline ferroptosis reducing fenton reaction substrates and lipid oxidizing enzyme cofactors [43,60]. Iron chelation in older worms increased lifespan of worms reducing frailty-induced ferroptosis [44].
**Ether lipids in** **ferroptosis**	Ether depletion through small molecule inhibition of AGPS led to cancer cell ferroptosis when treated with either DGLA or RSL3 [43]. In contrast, ether lipid synthesis inhibition that reduced PUFA levels led to rescue from ferroptosis [167].	Genetic depletion of ether lipids in *ads-1* strain worms are extremely sensitive to DGLA-induced ferroptosis, suggesting a protective role in ferroptosis [43].
